# Anesthetic management for abdominal aortic surgery in a patient with a left ventricular assist device: a case report

**DOI:** 10.1186/s40981-015-0011-8

**Published:** 2015-11-30

**Authors:** Koko Adachi, Toshihiro Wagatsuma, Takuya Shiga, Hiroaki Toyama, Masanori Yamauchi

**Affiliations:** Department of Anesthesiology and Perioperative Medicine, Tohoku University Graduate School of Medicine, 1-1 Seiryo-cho, Aoba-ku, Sendai, 980-0872 Japan

**Keywords:** Aortic cross-clamping, Left ventricular assist devices, Afterload, Preload, Anticoagulation

## Abstract

Left ventricular assist devices (LVAD) are a currently established destination and bridge therapy until cardiac transplantation; hence, this patient population continues to increase. Here, we present the first report of abdominal aortic cross-clamping (ACC) in a LVAD patient undergoing emergency aneurysm repair. Anticoagulation was continued pre-and intra-operatively to avoid pump thrombosis. The pumping function of the LVAD is highly dependent on both preload and afterload. In this case, abdominal ACC, which increases the afterload, did not significantly influence circulatory dynamics. However, when the abdominal ACC was released, mean atrial pressure (MAP) fell to 42 mmHg, because preload reduction due to massive bleeding (3532 g) secondary to anticoagulation and afterload reduction by abdominal ACC release combined to cause critical hypotension. Maintenance of MAP required rapid infusion and use of an alpha-adrenergic agent. Surgical and anesthesia times were 411 and 525 min, respectively. Total blood loss was 5389 g, respectively. The patient was discharged after 25 postoperative days with no major complications. ACC release, with its accompanying decrease in preload and afterload, and massive bleeding due to anticoagulation in these patients require careful management.

## Background

The presence of implanted left ventricular assist devices (LVAD) presents a dilemma when managing other serious medical problems in this patient population [1, 2]. Although there are some reports of anesthetic management in patients with LVAD undergoing non-cardiac surgery [3–8], this is the first report of abdominal aortic cross-clamping (ACC) performed in a patient with a LVAD, which is known to be sensitive to preload and afterload.

## Case presentation

Written informed consent was obtained from the patient for publication of this Case report. The patient was a 51-year-old male (height 171 cm, weight 60 kg). He had had cardiac resynchronization therapy with a defibrillator device implanted 2 years earlier, as well as a HeartMate II (Thoratec Co., Pleasanton, CA, USA.) implanted 6 months earlier for ischemic cardiomyopathy. Concurrent anticoagulation therapy included warfarin and aspirin. He was urgently admitted to the hospital on the day prior to surgery due to infected pseudoaneurysms of the bilateral common iliac arteries, which were deemed suitable for Y-shaped graft replacement. Preoperative transthoracic echocardiography displayed an ejection fraction of 5 %. As his PT-INR was 3.28, warfarin was discontinued and an infusion of heparin 2000 U/day was commenced 1 day before surgery.

**Fig. 1:**
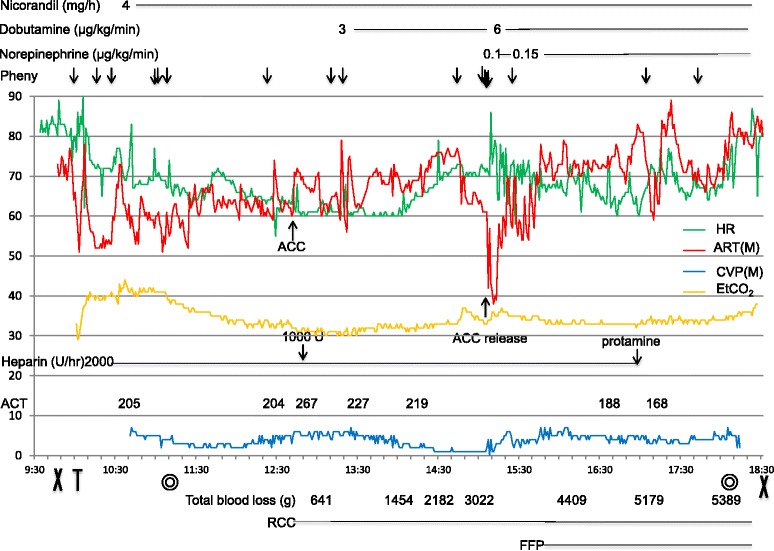
Anesthetic record. *Pheny* phenylephrine, *HR* heart rate, *ART* (*M*) mean arterial pressure, *CVP* (*M*) central venous pressure, *EtCO2* end-tidal carbon dioxide, *ACT* activated clotting time, *RCC* red cell concentrate, *FFP* fresh frozen plasma, *ACC* aortic cross-clamping, ☓ start and completion of anesthesia, *T* intubation, ◎ start and completion of surgery

In the operating room, the patient was monitored for ECG, pulse oximetry, arterial blood pressure, central venous pressure (CVP), transesophageal echocardiography (TEE), and bispectral index (BIS). The LVAD parameters were as follows: pump speed, 8200 rpm; calculated flow, 4–5 L/min; pump power, 5–6 W. The patient’s anesthetic record is shown in Fig. 1. Heart rhythm was a mixture of spontaneous and pacemaker rhythm (AAI, HR 60). Heparin 2000 U/h was continued during the surgery. Anesthesia was induced with fentanyl 100 μg, remifentanil 0.3 μg/kg/min, midazolam 10 mg, and rocuronium 60 mg, and maintained with propofol 3–4 mg/kg/h, remifentanil 0.1–0.3 μg/kg/min, and fentanyl 1300 μg. Hypotension was treated with intermittent boluses of phenylephrine (0.05–0.1 mg). When the infrarenal abdominal ACC was applied under the same LVAD conditions, MAP and CVP increased from 61 to 71 mmHg and from 5 to 6 mmHg, respectively. ACC time was 124 min. During ACC, bleeding, the source of which could not be pinpointed, continued. Before ACC release, the volume of blood lost was 3532 g, and the volumes of fluid (including colloid solutions) and red cell concentrate (RCC) infusions were 5400 and 1680 ml, respectively. When the ACC was released, MAP decreased to 42 mmHg, and a decrease in the size of both ventricles was observed on TEE, but the sucking phenomenon was not observed. The calculated flow of the LVAD also decreased to 3 L/min. We requested the surgeon to re-clamp the aorta and re-release it gradually. Simultaneously, acute massive transfusion, fluid infusion, and administration of phenylephrine (total 0.4 mg) were performed for maintenance of MAP. Further, an infusion of norepinephrine (0.1–0.15 μg/kg/min) was started. With this, MAP increased to 60 mmHg, and calculated LVAD flow returned to 5 L/min within 15 min. At this time, the values of pH and base excess were 7.354 and −2.4, respectively. Thereafter, the heparin infusion was stopped, and 10 mg of protamine was administered. Surgical and anesthesia times were 411 and 525 min, respectively. Total blood loss and urine output were 5389 g and 1507 ml, respectively. The volumes of fluid, RCC and fresh frozen plasma (FFP) infusions were 7200, 2520, and 960 ml, respectively.

The patient was transferred to the intensive care unit and extubated the next day. He was discharged 25 days later with no major complications.

The hemodynamic management of LVAD patients during non-LVAD surgery is important because the pump function depends on both preload and afterload [9]. Continuous flow LVADs are up to three times more sensitive to a change in afterload compared with normal heart function [10]. Therefore, in this case, we were anxious about the hemodynamics at the time of ACC application and release. However, application of the ACC did not result in a significant change in MAP. This was assumed to be due to the venodilatation and relative dehydration resulting from anesthesia induction. Another reason for this may have been the fact that HeartMate II is an axial pump, and axial pumps are less afterload sensitive than centrifugal pumps [11]. On the other hand, when the ACC was released, MAP decreased precipitously. This could be because ACC release pathophysiologically decreased both afterload and venous return [12]. It has been reported that HeartMate II, in particular, demonstrates increased preload sensitivity in the low-afterload region as compared to other LVADs [11]. In this case, blood loss increased rapidly 30 min before ACC release, together with a fall in CVP. Ideally, we should have started adequate preloading and infusion of an alpha-adrenergic agent before ACC release. Moreover, a pulmonary artery catheter should have been used to monitor actual cardiac output, since the calculated flow shown on the system console may not always have a high degree of fidelity with the patient’s true cardiac output [13]. It is possible to perform this surgery using cardiopulmonary bypass. The advantage of cardiopulmonary bypass is that to achieve stable circulatory dynamics, and the disadvantage is to increase operative stress.

LVAD patients chronically require anticoagulation to avoid pump thrombosis [3, 14]. In this case, because ACC required systemic heparinization, heparin was continued. Hemostasis, on the other hand, required discontinuation of heparin, administration of protamine, and FFP transfusion. A previous report suggests that the risk of bleeding due to impaired platelet aggregation in HeartMate II-treated patients may be significant [15]. Hence, the anesthetic management of LVAD patients should include a plan against massive bleeding preoperatively using FFP and platelet concentrates, while balancing intra-operative bleeding with pump thrombosis. Anticoagulant management is likely to be even more challenging when these patients present for emergency surgery.

RV dysfunction is another potential cause of reduced LVAD output. Thus, maintaining RV function in LVAD patients undergoing surgical procedures is extremely important. This can be achieved pharmacologically by a combination of inotropes and RV afterload reducers. We achieved this by using dobutamine, and avoiding increases in pulmonary vascular resistance (e.g., due to hypoxemia, hypercarbia, and acidosis).

## Conclusions

This is the first report of abdominal ACC performed in a LVAD patient sensitive to preload and afterload. Application of the ACC did not significantly affect circulatory dynamics, although its release caused a marked decrease in MAP. ACC release, with its accompanying decrease in preload and afterload, and massive bleeding due to anticoagulation in these patients require careful management.

## Consent

Written informed consent was obtained from the patient for publication of this case report and any accompanying images. A copy of the written consent is available for review by the Editor-in-Chief of this journal.
